# Pediatric Post-Discharge Mortality in Resource Poor Countries: A Systematic Review

**DOI:** 10.1371/journal.pone.0066698

**Published:** 2013-06-25

**Authors:** Matthew O. Wiens, Shane Pawluk, Niranjan Kissoon, Elias Kumbakumba, J. Mark Ansermino, Joel Singer, Andrew Ndamira, Charles Larson

**Affiliations:** 1 Department of Pediatrics, Mbarara University of Science and Technology, Mbarara, Uganda; 2 School of Population and Public Health, University of British Columbia, Vancouver, Canada; 3 Faculty of Pharmaceutical Sciences, University of British Columbia, Vancouver, Canada; 4 Department of Pediatrics, BC Children's Hospital and University of British Columbia, Vancouver, Canada; 5 Department of Anesthesia, BC Children's Hospital and University of British Columbia, Vancouver, Canada; 6 Centre for International Child Health, Child and Family Research Institute, BC Children's Hospital and University of British Columbia, Vancouver, Canada; University of Utah School of Medicine, United States of America

## Abstract

**Objectives:**

Mortality following hospital discharge is an important and under-recognized contributor to overall child mortality in developing countries. The primary objective of this systematic review was to identify all studies reporting post-discharge mortality in children, estimate likelihood of death, and determine the most important risk factors for death.

**Search Strategy:**

MEDLINE and EMBASE were systematically searched using MeSH terms and keywords from the inception date to October, 2012. Key word searches using Google Scholar™ and hand searching of references of retrieved articles was also performed. Studies from developing countries reporting mortality following hospital discharge among a pediatric population were considered for inclusion.

**Results:**

Thirteen studies that reported mortality rates following discharge were identified. Studies varied significantly according to design, underlying characteristics of study population and duration of follow-up. Mortality rates following discharge varied significantly between studies (1%–18%). When reported, post-discharge mortality rates often exceeded in-hospital mortality rates. The most important baseline variables associated with post-discharge mortality were young age, malnutrition, multiple previous hospitalizations, HIV infection and pneumonia. Most post-discharge deaths occurred early during the post-discharge period. Follow-up care was examined in only one study examining malaria prophylaxis in children discharged following an admission secondary to malaria, which showed no significant benefit on post-discharge mortality.

**Conclusions:**

The months following hospital discharge carry significant risk for morbidity and mortality. While several characteristics are strongly associated with post-discharge mortality, no validated tools are available to aid health workers or policy makers in the systematic identification of children at high risk of post-discharge mortality. Future research must focus on both the creation of tools to aid in defining groups of children most likely to benefit from post-discharge interventions, and formal assessment of the effectiveness of such interventions in reducing morbidity and mortality in the first few months following hospital discharge.

## Introduction

Acute diseases leading to death and significant morbidity continue to plague children in resource limited areas of the developing world disproportionately. About 70% of deaths are due to infectious diseases. While effort has been made to address diagnosis and treatment during the acute episode, care following discharge from hospital is an important aspect of management that is often neglected by both policy makers and health researchers. Reasons for this neglect are likely multifactorial and include a tremendous burden and high costs to provide care for acute illness, which in regions with limited resources poses significant system challenges. Furthermore, failure to recognize and document the burden of post-discharge morbidity and mortality contributes to a lack of awareness by health care workers of potentially avoidable adverse outcomes. Therefore, the attempt to improve care following discharge is viewed as a low priority by both health care workers and policy makers. Lack of attention to post-discharge issues has tremendous adverse implications because the scant available evidence strongly suggests that in developing countries post-discharge deaths may be of similar (or higher) magnitude than deaths during hospitalization [1]–[5]. These data suggest that improved discharge planning and post-discharge care has the potential to decrease the need for readmission, and to significantly decrease morbidity and mortality. This discharge process will be an important step in achieving the fourth millennium development goal (MDG) of a two thirds reduction in under-five mortality [6].

While current evidence clearly points to the significant burden of post-discharge mortality, the estimates of this burden vary widely between studies. Factors such as age, co-morbidities, disease severity, healthcare resources, length of follow-up, and social disparities are likely to play a significant role in determining outcome. Using the guidelines set forth by the Meta-analysis of Observational Studies in Epidemiology Group [7] we sought to compile all studies investigating pediatric post-discharge mortality in low income countries. Our primary objective was to describe the rates of mortality following medical discharge in children and identify the risk factors which are associated with post-discharge mortality.

## Methods

### Search Strategy

We conducted a systematic computerized search from the inception date (1946 in MEDLINE and 1974 in EMBASE) to October, 2012 to identify all potentially eligible studies. One investigator (SP) trained in database searching independently carried out an initial systematic search. A study was defined as an analysis of post-hospitalization mortality in a pediatric population. We applied the following algorithm in both medical subject heading (MeSH) and free text words. In MEDLINE, the MeSH terms “follow-up studies,” “hospitalization,” OR “longitudinal studies” were combined with “developing countries,” “Africa,” “Bangladesh,” “Haiti,” “Afghanistan,” “Yemen,” “Papua New Guinea,” “Myanmar,” “Pakistan,” OR “Solomon Islands.” MeSH terms were exploded where appropriate. The MeSH term “Africa” included the names of all African countries when exploded. Free text words including “post-discharge mortality” and “long-term outcomes” were also used to increase capture of relevant articles. In EMBASE, the MeSH terms “follow-up,” “hospitalization,” OR “longitudinal study” were combined with “developing country,” “Bangladesh,” “Haiti,” “Afghanistan,” “Yemen,” “Papua New Guinea,” “Burma,” “Pakistan,” “Solomon Islands” OR “Melanesia” AND “Pediatrics.” Free text word “Burma” was also included in the search as this was not a MeSH term. Google Scholar™ was also searched and references of relevant publications were reviewed to identify any articles not captured during initial search. All retrieved articles were independently reviewed by a second author (MW) to determine if they met inclusion criteria.

#### Inclusion Criteria

Studies were included if: (i) they presented original data from randomized-controlled trials, cohort studies, or retrospective analyses; (ii) the data on post-discharge mortality in pediatric patients of any age was clearly defined and length of follow-up was reported; (iii) data was collected from pediatric patients living in developing countries. Developing countries were defined for the purposes of this review as those countries currently classified by the United Nations Development Program (UNDP) as having a low Human Development Index (HDI) [8].

#### Exclusion Criteria

Studies were excluded if: (i) there was no pediatric data or pediatric data could not be differentiated for adult data; (ii) there was no post-hospital discharge information or patients were not discharged from a hospital setting; (iii) discharge was following a non-admission (i.e. following birth); (iv) studies represented a surgical population since post-discharge care following surgery would likely be very different from care following acute illness and; (v) if the study was unpublished, published in a language other than English or if published only in abstract form.

### Data Collection and Quality Assessment

Data was collected systematically onto a computerized spreadsheet developed *a priori*, which included study name and year of publication, primary author, country, number of participants, age of study population, reason for hospital admission (either medical or surgical), in-patient mortality, post-discharge mortality, post-discharge hospitalization, post-discharge observation period and risk factors of mortality. When the study was reported as the result of a randomized controlled trial, data from both the control arm and intervention arm was collected and reported either individually or as a combined estimate when appropriate.

While a validated quality scoring system for studies on post-discharge mortality has not been developed, several variables likely to contribute to study quality were collected and reported including: proportion of subjects successfully followed-up, method of follow-up, study design and presence of an intervention.

### Analysis

Since significant heterogeneity between studies was observed studies were not pooled. Studies were organized based on underlying etiology in the study sample. Descriptive statistics were generated using Microsoft Excel (Redmond, WA).

## Results

**Figure 1:**
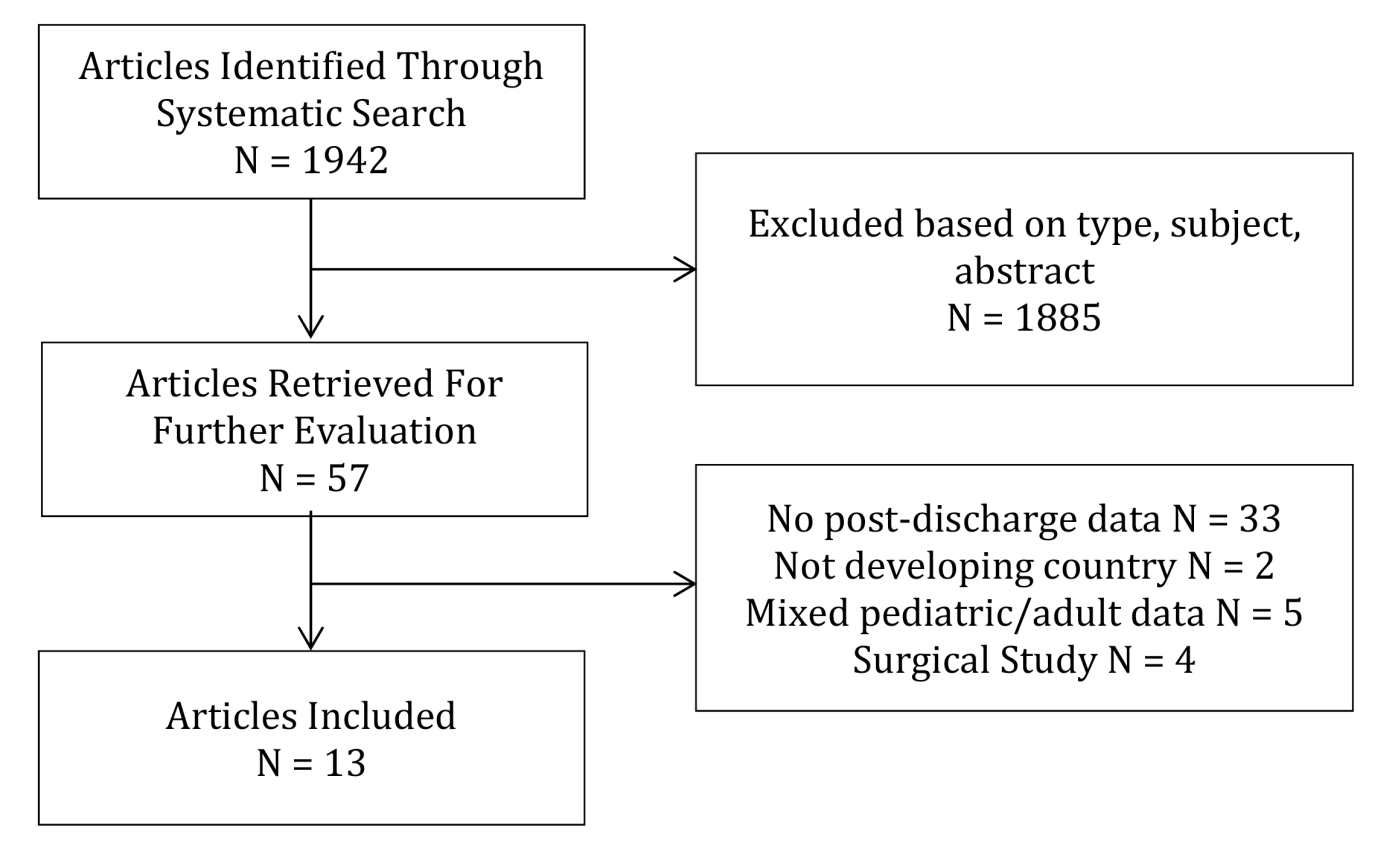
Flow diagram of the search for identifying and selecting studies of post-discharge mortality.

Thirteen studies met both inclusion and exclusion criteria and were included in the final analysis; four randomized controlled trials [3], [4], [9], [10], four prospective cohort studies [1], [11]–[13], three retrospective cohort studies [14]–[16], and two case-control studies with longitudinal follow-up of cases and/or controls [2], [17] (**Figure 1**). No studies were excluded based on language of publication. Four studies were from Bangladesh, three from Guinea-Bissau, two from both Kenya and Malawi, one from Tanzania, one from the Democratic Republic of Congo and one from The Gambia. The pediatric populations which the studies represented varied widely by study according to both age and underlying disease state. The disease states represented included four studies of all children admitted to hospital; two studies of children admitted with malaria; three studies of children admitted with diarrhea; three studies of children admitted with pneumonia; one study of children admitted with anemia; and one of children admitted with malnutrition (**Table 1**). Rates of post-discharge mortality varied widely between studies (1% – 18%) as did the durations of post-discharge follow-up (approximately 28 days – 5 years). Seven studies reported the approximate proportion of children surviving at various time-points during follow-up and reported that most children who died did so during the early phase of follow-up (**Table 2**). Risk factors for post-discharge mortality varied significantly between studies but the most important included young age, malnutrition, multiple previous discharges, HIV infection and pneumonia (**Table 3**).

**Table 1:** Characteristics of all included studies.

Ref.	Design			Period		Country	Age Range		Population		Locale*			N		FU Proportion		FU Method			FU Times		IP Mortality	PD Mortality		PD deaths in hospital			PD hospitalization				Obs. Period
**Studies of all pediatric hospital admissions**																																	
(14)	Retrospective Cohort			1991–1996		Guinea-Bissau	(81%<5y)		All Admits		NR			3373		NA		Surveillance			NA		12.10%	6.10%		23.10%			11.60%				12 m
(15)	Retrospective Cohort			2003–2008		Kenya	0–15y		All Admits		Mixed			14,971		NA		Surveillance			NA		NR	4.50%		NR			NR				12 m
(1)	Prospective Cohort			1991		Kenya	0–5y		All Admits		Mixed			1223		96%		CV & HV			4, 8 w		10%	13%		NR			NR				8 w
**Studies of admissions secondary to anemia**																																	
(2)	CC with longitudinal FU			2002–2004		Malawi	6–60 m		Anemia (Hg<50g/L)		Mixed		377			82.20%		CV		1, 3, 6, 12,18 m			6.4%	11.6%		NR ("most")		17.20%			18 m		
							7–60 m		Any other condition		Mixed		373			80.40%		CV		1, 3, 6, 12,18 m			0.0%	2.7%		NR ("most")		9.40%			18 m		
**Studies of admissions secondary to malaria**																																	
(9)		RCT	2006–2009		Malawi		4–59 m	IPTpd		Mixed		706			95%		PCD		1, 2, 3, 6m			NA		2.6%		NR			19%			6 m	
							5–59 m	Placebo		Mixed		708			95%		PCD		1, 2, 3, 6m			NA		2.4%		NR			19%			7 m	
(10)		RCT	2004–2006		Guinea–Bissau		3–60 m	Severe malaria		NR		951			95%		CV & HV		28 d after admission			7.20%		2.0%		NR			NR			28d after admission	
**Studies of admissions secondary to diarrhea**																																	
(12)	Prospective cohort			1979		Bangladesh	3 m–3y		Diarrhea		Rural			551		NR		NR			NR		NA	4.2%		NR			NR				12 m
(11)	Prospective cohort			199–1992		Bangladesh	1–23 m		Diarrhea		Urban			500		85% at 6w, 80% at 12w		HV			6, 12 w		NA	7%		53%			NR				12 w
(16)	Retrospective cohort			1983–1983		Bangladesh	24–72 m		Diarrhea		Urban			74		93%		HV			Approx. 4m		NR	2.9%		NR			NR				Approx. 4m
**Studies of admissions secondary to pneumonia**																																	
(3)	RCT			1993–1997		Tanzania	6–60 m		Pneumonia		NR		687			89%		CV		Monthly			3%	10%	NR		NR			24 m			
(4)	RCT			2006–2008		Bangladesh	2–59 m		Severe pneumonia		Urban		180			90%		CV		Every 2 w			0%	1%	NR		6%			3 m			
(17)	CC with longitudinal FU			1992–1997		The Gambia	0–5 m		LRTI, SpO2 <90		Mixed		83			64%		CV & HV		Once			NR	15%	NR		NR			mean 41 m			
									LRTI, SpO2 ≥90		Mixed		107			61%		CV & HV		Once			NR	6%	NR		NR			mean 34 m			
**Studies of admissions secondary to malnutrition**																																	
(13)	Prospective cohort			1970		DRC	NR		PEM		NR		171			76		CV			Annually		NA	18%		NR		NR			5y		

**Table 2:** Timing of post-discharge deaths.

Study	Population	Obs. period	Time-point for 50% of PD deaths	Other mortality statistic
(11)	1–23 m with diarrhea	12 w	10d	
(12)	3 m-3y with diarrhea	12 m	30d	
(14)	All pediatric admissions	12 m	60d	
(1)	0–5y all admissions	8 w		82% PD deaths at 4 w
(3)	6–60 m with pneumonia	24 m		80% PD deaths at 12 m
(13)	malnutrition	5y		>50% PD deaths at 1y
(2)	6–60 m with anemia	18 m		71% at PD deaths at 6 m*

**Table 3:** Risk factors for post-discharge mortality.

Ref.	Population	Risk factors for post-discharge mortality	Adjusted RR or HR (95% CI)
**Studies of all pediatric admissions**			
(15)	All admissions	Age 1–5 m	1.34 (0.93–1.92)
		Age 6–11 m	0.82 (0.57–1.18)
		Age 2–5 y	0.57 (0.36–0.90)
		Weight-for-age Z score <−3	3.42 (2.5–4.68)
		Weight-for-age Z score <−4	6.53 (4.85–8.80)
		Parasitemia	0.45 (0.29–0.71)
		Hypoxia	2.30 (1.64–3.23)
		Bacteremia	1.77 (1.15–2.74)
		Jaundice	1.77 (1.08–2.91)
		Hepatomegaly	2.34 (1.60–3.42)
		Hospitalization >13d	1.83 (1.33)
		1 prior discharge	2.83 (2.04)
		2 prior discharges	7.06 (4.09–12.21)
		≥3 prior discharges	23.55 (10.70–51.84)
		Mild pneumonia	2.30 (1.00–5.28)
		Severe pneumonia	1.37 (1.05–1.79)
		Very severe pneumonia	4.09 (2.25–7.46)
		Severe malnutrition	4.37 (2.73–7.01)
		Meningitis	2.29 (1.57–3.32)
		Sick young infant	2.67 (1.98–3.58)
		HIV	2.22 p = 0.19
		Absconded	2.06 p = 0.95
(14)	All admissions	Mother educated	0.74 (0.55–0.99)
		Discharged against medical advice	8.51 (5.32–13.59)
		Anemia (vs. malaria)	1.97 (1.07–3.63)
		Diarrhea (vs. malaria)	1.82 (1.21–2.74)
		Pneumonia (vs. malaria)	0.98 (0.65–1.51)
		Measles (vs. malaria)	0.77 (0.36–1.64)
		≥5y (vs. 1–12 m)	0.15 (0.07–0.30)
		4–5y (vs. 1–12 m)	0.23 (0.10–0.59)
		3–4y (vs. 1–12 m)	0.14 (0.06–0.35)
		2–3y (vs. 1–12 m)	0.52 (0.33–0.81)
		1–2y (vs. 1–12 m)	0.82 (0.59–1.13)
		Neonatal (vs. 1–12 m)	0.69 (0.31–1.55)
**Studies of anemia admissions**			
(2)	Anemia (Hg<50 g/L) admissions	Increase in age (months)	0.92 (0.87–0.97)
		Rural (vs. urban)	1.63 (0.63–3.52)
		Male (vs. female)	1.54 (0.68–3.52)
		Parental unemployment	4.15 (1.61–10.74)
		Splenomegaly	0.36 (0.16–0.80)
		HIV	10.49 (4.05–27.20)
		Bacteremia	2.17 (0.84–5.64)

Ref.	Population	Risk factors for post-discharge mortality	Adjusted RR or HR (95% CI)
Studies of diarrhea admissions			
(11)	Diarrhea admissions	Age (1–6 m vs. 6–24 m)	4.57 (2.90–7.18)
		Sex (Female)	1.73 (1.14–2.65)
		Maternal Education (none vs. ≥1y)	2.12 (1.37–3.28)
		Child was not breastfed	2.35 (1.44–3.84)
		Weight-for-age median <60% vs. ≥60%	1.04 (0.57–1.89)
		Length-for-age median <85% vs. ≥85%	2.97 (1.43–6.16)
**Studies of pneumonia admissions**			
(17)	Pneumonia admissions	Weight-for-age Z-Score <−2	3.2 (1.03–10.29)
		Length of stay	SS* (RR not reported)

### Studies of all Hospital Admissions

Three studies (two from Kenya and one from Guinea-Bissau) included all children regardless of admission diagnosis. The first Kenyan study, a prospective cohort study conducted in 1991, enrolled 1223 children between 0 and 5 years of age at the time of admission and followed these children until 8 weeks following discharge [1]. During this period 10% of children died during their hospitalization and 13% of children who were discharged died. The second retrospective study was conducted in 2011 in Kenya and examined 12 month post-discharge mortality between the years 2003 and 2008 among children 0 to 15 years of age [15]. Using a pre-existing surveillance system they found that mortality was 4.5%. The main strength of this study was the large number of study subjects (14,971) and the detailed analysis of post-discharge mortality risk factors. The most notable risk factors for post-discharge mortality was previous hospitalization with three or more discharges producing a hazard ratio of 23.5 (95%CI 10.70-51.84) and 2 previous discharges producing a statistically significant hazard ratio of 7.06. Very severe pneumonia and very low weight for age scores also produced statistically significant hazard ratios of 4.09 and 6.53, respectively (**Table 2**). The study from Guinea-Bissau was also a retrospective cohort study based on surveillance data. It followed children who were primarily below 5 years of age and found that in-hospital mortality was approximately 12% while post-discharge mortality was approximately 6% [14]. The primary risk factors for post-discharge mortality were discharge against medical advice (RR 8.51, 95% CI 5.32-13.59), anemia and diarrhea (RR 2.0 and 1.8, respectively).

### Malaria Studies

Two studies of children with malaria, both of which were randomized trials, were identified. The first study, conducted in Malawi between 2006 and 2009, randomized children with severe malaria to receive intermittent preventative therapy (IPTpd) or placebo following hospital discharge [9]. Over the course of six months of follow-up, similar numbers of children in both the IPTdp and placebo groups died (2.6% vs. 2.4%, respectively). Nearly 20% of children discharged required subsequent hospitalization. The second study, conducted in Guinea-Bissau between 2004 and 2006, examined the effect of a financial incentive to health care workers to improve hospital treatment of acute malaria [10]. Within 4 weeks of admission overall mortality among both groups was approximately 8.7% with a 7.2% in-hospital mortality and 2% post-discharge mortality rate. Since the period of follow-up was calculated from admission, no specific length of follow-up was conducted. Overall it was approximately 3 weeks as the mean length of stay was approximately 1 week.

### Diarrhea Studies

Three studies investigating outcomes following diarrhea were identified, all of which were conducted in Bangladesh between the late 1970 s and the early 1990 s. The most recent study conducted between 1991 and 1992 enrolled 500 urban children who were admitted and treated for diarrhea [11]. With 80% follow-up at 12 weeks post-discharge they found that post-discharge mortality was 7% and that approximately half of these deaths occurred during a re-admission (non-fatal re-admissions were not reported). This study conducted verbal autopsies and found that the primary cause of death was a diarrheal disease in 69% of cases and an acute respiratory disease in 31% of cases. Given the relatively low proportion of follow-up it is likely that the actual post-discharge mortality rate was higher. This study reported that young age, short stature for age, lack of breastfeeding, low maternal education, and female sex were all predictors of post-discharge mortality. The remaining two studies were from 1979 and 1983 and of relatively poor methodological quality. The post-discharge mortality rates were approximately 4% and 3%, respectively, and the hospital course was not described [12], [16].

### Pneumonia Studies

Three studies of outcomes following pneumonia were identified, two of which were randomized trials. The first study was a secondary analysis of a trial of vitamin A supplementation in children 6–60 months of age with pneumonia [3]. This study found that in-hospital mortality was 3% and post-discharge mortality was 10% after 24 months. Risk factors for post-discharge mortality were not calculated, but HIV infection (3.92 95%CI 2.34–6.55), young age (3.70, 95%CI 1.72–7.95), unclean water source (2.92, 95%CI 1.03–8.30), severe anemia (2.55 95%CI1.13–5.77), severe pneumonia (2.47, 95%CI 1.59–3.85), and nutritional indicators such as stunting (2.12, 95%CI 1.31–3.42) were associated with increased overall mortality (in-patient and post-discharge). The second study was a randomized controlled trial of in-patient versus out-patient management of severe pneumonia in Bangladesh [4]. There were no deaths during hospitalization and only 1% mortality in 180 children who were followed for 3 months following discharge suggesting that this was a low-risk group of patients. The final study was a follow-up study of a case-control study assessing predictors of hypoxemia in Gambian children. The initial study, conducted between 1992 and 1994, enrolled 190 children admitted with a lower respiratory infection. Follow-up was conducted between 1996 and 1997 during which 15% of hypoxemic children (SpO2 <90%) and 6% of non-hypoxemic children died. Differences in mean length of follow-up were observed (41 months in hypoxemic group vs. 34 months in non-hypoxemic group) and a poisson regression showed that mortality rates were not statistically significantly different. However, this was not an appropriate analysis since this assumes a constant hazard over time, an assumption unlikely to be correct for post-discharge mortality. Similar to other studies, low weight for age Z-scores during admission were associated with higher post-discharge mortality rates (RR 3.2 95% CI 1.03–10.29).

### Anemia Studies

One study aiming to determine the short and long term effects of severe anemia in children conducted in Malawi in 2008 was identified [2]. This study was the longitudinal part of an earlier case-control study and had two arms (cases and controls) which where independently followed for 18 months after discharge. In the anemia arm (cases) 377 children were enrolled of whom 6.4% died in hospital and 11.6% died following discharge over the course of 18 months. In the non-anemia arm (controls), consisting of children with any condition other than anemia, none of the 373 children died in hospital and 2.7% died following discharge. This study had a low rate of follow-up (approximately 80%) relative to the other studies. In the anemic group, HIV, bacteremia, and nutritional deficiency (stunting/wasting) were more common in those who died following discharge compared to survivors, however no formal analysis was done in this regard.

### Malnutrition Studies

One study assessed survival following successful hospital treatment of protein energy malnutrition in the Democratic Republic of Congo (formerly Zaire) [13]. This study followed 171 children for 5 years after discharge and found that 18% died. The follow-up rate over this time was 76%. Mortality after 1 year was 10% indicating that most deaths occurred relatively early. While young age was predictive of post-discharge death (mean age of 26 vs. 59 months in dead and surviving children, respectively) neither weight-for-age, height-for-age, length of stay, or degree of hypoalbuminuria was associated with death at 1 year or 5 years following discharge.

## Discussion

Thirteen studies that reported post-discharge mortality rates were identified. Studies varied in design, length of follow-up, location and in study population. The majority of studies were from African countries. In these studies we found a consistent trend of mortality rates similar to those seen in hospital. Of the six studies that reported both in-patient and post-discharge mortality, four reported mortality rates higher following discharge than during hospitalization.

The term “post-hospital syndrome” has recently been introduced and describes an acquired, transient period of vulnerability following discharge [18]. Not only does the acute (and sometimes chronic) illness contribute to derangements in normal physiologic function, other stressors such as sleep deprivation, poor nutrition, pain and adverse effects of medications contribute to a state in which the patient is more vulnerable to decline, even following recovery of the initial acute condition. Sepsis, the most common cause of death among children in developing countries [19], [20], is known to cause significant losses in adaptive immunity [21], perhaps contributing to the significant burden of post-discharge mortality observed.

Ideally, all children discharged from hospital should be followed-up to ensure identification of children suffering re-emergence of an acute illness; however in an already over-burdened health system this is neither feasible nor cost-effective. Therefore, the identification of risk factors for post-discharge mortality is an important starting point for interventions aiming to reduce morbidity and mortality following discharge. In those studies which identified such risk factors, nutritional indicators (such as weight–for-age), young age, and previous hospitalizations as well as disease specific factors such as HIV infection and pneumonia were consistently associated with a poor prognosis following discharge. The only study identified which actively addressed post-discharge mortality built upon previous research indicating anemia was an important predictor of mortality after discharge. Unfortunately, however, the intervention of providing malaria prophylaxis did not substantially reduce 6 month post-discharge mortality. The timing of post-discharge deaths is also an important consideration since this may aid in determining the period during which post-discharge interventions should be applied. While the duration of follow-up varied significantly between studies (28 days – 5 years), the probability of death was substantially higher during the first several months, indicating that post-discharge interventions during this period may offer the highest probability of success.

The integrated management of childhood illness (IMCI) program developed by the World Health Organization (WHO) is an attempt to compile the best available evidence for treatment of common pediatric diseases and facilitate the uptake of a standardized approach to these diseases in resource poor countries [22]. Even though significant focus of the IMCI has been placed on both inpatient and outpatient treatment there is a general lack of evidence based recommendations on the prevention of post-discharge morbidity and mortality. Formal recognition of the morbidity and mortality following hospital discharge, and its associated risk factors is required. The recent post-discharge surveillance study from Kenya analyzed the utility of identifying children with any one of several individual risk factors to determine the sensitivity and specificity of identifying children likely to die following discharge [15]. This study found that the presence of either low weight-for-age score, hospitalization greater than 13 days, hypoxia, bacteremia, hepatomegaly, or jaundice would identify 33% of discharges and 47% of post-discharge deaths. While this research can be used to better improve post-discharge care, significant numbers of deaths following discharge would still not be identified. Furthermore, limited resources for risk factor determination (such as blood culture) would make this process difficult to implement in many health centers throughout Africa. A new research approach specific to the identification of easily measured risk factors for use in a simple clinical prediction tool developed and validated for use in poorly resourced health centers could prove very useful. Furthermore, defining the population in whom such a prediction tool would be implemented in is also important as significant differences exist between patient groups to warrant different prediction tools (such as children with infectious diseases vs. children without infectious diseases). Once such tools are validated they could be incorporated into guidelines such as the IMCI to better improve post-discharge initiatives.

In addition to specific risk factors for, and timing of, post-discharge mortality, we also observed that in several studies many children who died did not die during a re-admission but rather died at home [11], [14]. Although barriers to returning to hospital were not discussed in any of the studies, factors such as transportation costs, care costs and poor care may have contributed to this. Studies to identify specific barriers at the community level among parents of recently discharged children could help drive effective interventions to improve health seeking behavior. Technological innovations such as the utilization of cellular technology may assist in identification of sick children in need of referral. Volunteer health workers have been utilized in a unidirectional manner (home-to-hospital) in many settings to identify children requiring community level treatment or referral to referral centers [23] Use of these health workers for discharge referrals would decrease resources required for effective follow-up and referral in cases of disease emergence. Efforts by policy makers and global health funding organizations to overcome barriers such as these are required if health seeking behavior following hospital discharge is to improve.

One limitation of this review was that the studies that were identified often did not have post-discharge mortality as a primary outcome. It is therefore possible that other similar studies, further removed from the search terms used, were not identified. However, the systematic search utilized was intended to be sufficiently broad to identify most of such studies. Another limitation was that several studies had follow-up rates below 90%. It is unlikely, however, that the reported mortality rates would be lower since losses to follow-up likely represent a more vulnerable population with higher rates of post-discharge mortality. A further limitation was the lack of a valid quality scoring system. As most studies were not specifically designed to assess post-discharge mortality a scoring system based on general study features could also not be created. The reason was that most of the study features in various statements (CONSORT, STROBE etc.) are for determining validity for drawing specific inferences according to the objectives of the study. Presence (or lack) of these characteristics does not necessarily mean that inferences for post-discharge mortality estimates are good (such as blinding in an RCT).

## Conclusions

Pediatric post-discharge mortality is a significant and generally unrecognized problem in developing countries. While several characteristics are strongly associated with post-discharge mortality, no validated tools are available to aid health workers or policy makers in the systematic identification of children at high risk of post-discharge mortality. Global health policy and research must focus on both the creation of tools to aid in defining groups of children most likely to benefit from post-discharge interventions, formal assessment such interventions, followed by the scale-up of effective interventions.
